# Radiological Markers of the Olfactory Cleft: Relations to Unilateral Orthonasal and Retronasal Olfactory Function

**DOI:** 10.3390/diagnostics10110989

**Published:** 2020-11-23

**Authors:** David T. Liu, Ursula Schwarz-Nemec, Bertold Renner, Christian A. Mueller, Gerold Besser

**Affiliations:** 1 Department of Otorhinolaryngology, Head and Neck Surgery, Medical University of Vienna, 1090 Vienna, Austria; david.liu@meduniwien.ac.at (D.T.L.); gerold.besser@meduniwien.ac.at (G.B.); 2 Department of Biomedical Imaging and Image-Guided Therapy, Medical University of Vienna, 1090 Vienna, Austria; ursula.schwarz-nemec@meduniwien.ac.at; 3 Institute of Experimental and Clinical Pharmacology and Toxicology, Universität Erlangen-Nürnberg, 91054 Erlangen, Germany; bertold.renner@tu-dresden.de; 4 Institute of Clinical Pharmacology, Medical Faculty Carl Gustav Carus, Technische Universität Dresden, 01307 Dresden, Germany

**Keywords:** olfaction, chronic rhinosinusitis, Lund-Mackay, retronasal, anosmia, computed tomography, olfactory cleft, candy smell test, smell, flavor

## Abstract

The opacification of the olfactory cleft (OC) has been associated with birhinal orthonasal olfaction in patients with chronic rhinosinusitis (CRS). The aim of this study was to determine the associations between monorhinal and birhinal orthonasal, and retronasal olfaction with radiological markers of the OC in a cohort of patients with CRS. Results were analyzed in a CRS-cohort including 13 patients with nasal polyposis (CRSwNP) and 12 patients with non-eosinophilic CRS (non-eCRS). Monorhinal and birhinal orthonasal olfactory function, and OC-air volume were higher in non-eCRS compared CRSwNP. OC-opacification was also higher in CRSwNP compared to non-eCRS. In the entire CRS-cohort, those with higher OC-opacification showed significantly lower orthonasal and retronasal olfactory test results compared to those with lower OC-opacification across all three coronal planes. Similarly, higher unilateral OC-opacification was also associated with lower ipsilateral orthonasal olfactory function. Correlation analysis further revealed a positive correlation between monorhinal and birhinal orthonasal olfaction with ipsilateral and overall OC-air volume. Likewise, birhinal and monorhinal orthonasal, and retronasal olfactory test results correlated negatively with the overall and ipsilateral Lund-Mackay scores. Monorhinal and birhinal orthonasal, and retronasal olfactory function were lower in CRS patients with higher ipsilateral and overall OC-opacification compared to those with lower OC-opacification.

## 1. Introduction

Olfactory dysfunction (OD) is an important condition with significant impact on quality of life (QOL), which is quite common in the general population with a prevalence of 15–25% [1,2,3,4,5]. OD is even more important in certain subsets of patients, such as those with inflammatory sinus disease, for example chronic rhinosinusitis (CRS), in whom an impairment of the sense of smell is one of the four major symptoms and may be prevalent in up to 84% of cases [6]. This cardinal sign may often go unnoticed during clinical assessment (if not tested comprehensively), as the degree of complaint can be less pronounced in a gradual (as in sinonasal disease) versus a sudden onset (as in posttraumatic OD) [2,7]. Despite this high prevalence, little is known about possible radiological predictors of olfactory function and their clinical relevance.

Volatile molecules can reach the olfactory epithelium (which is embedded in the mucosa of the olfactory cleft, but also beyond [8]) either through both nostrils (orthonasal), or through the pharynx (retronasal). Retronasal olfaction contributes to our multisensory flavor perception to a large extent [9]. Numerous tools are available for testing the orthonasal route whereas tests for retronasal olfaction still need higher acceptance [2,10,11]. In a clinical context, orthonasal olfaction is usually not tested for each nostril separately (monorhinal or lateralized), but birhinally. This routinely used birhinal approach is well reasoned, considering personnel needed for testing. Noteworthy, unilateral-based olfactory testing may uncover relevant side differences in up to 20% [12]. Nevertheless, this comprehensive approach to olfaction is rarely considered in clinical studies, which possibly distorts findings on associations of e.g., anatomical or inflammatory differences and olfaction [13,14,15,16].

Chemosensory testing remains the gold standard to evaluate olfactory function in patients with OD. It has been shown that self-reported olfactory function is inconsistent with objective test results; hence, other predictors such as nasal endoscopy scores of the olfactory cleft (OC) have been considered [17,18,19,20,21]. However, since nasal septal deviations are common among the general population, reliable endoscopic evaluations of the OC may not be constantly feasible [22]. For this reason, clinicians may benefit from additional information obtained by radiological markers.

Previous studies have found evidence that an OC-specific radiologic grading scale can be associated with olfactory function [23,24]. More precisely, the opacification of the OC was found to be inversely correlated with all three olfactory dimensions, including odor threshold, discrimination, and identification for the phenotype of CRS with nasal polyposis (CRSwNP), whereas this correlation was only found to be significant for odor identification in the second phenotype of CRS without nasal polyps (CRSsNP) [25,26,27,28,29]. However, possible associations of unilateral olfaction and OC-opacification, and the impact of this imaging biomarker on retronasal olfaction have yet to be elucidated, which was the aim of the present investigation.

## 2. Materials and Methods

### 2.1. Subjects

Adult patients (older 18) were recruited from the Department of Otorhinolaryngology at the Medical University of Vienna between October 2018 and June 2019. Olfactory data were collected one day prior to surgery in a prospective study on various rhinosurgical procedures [30]. All paranasal CT examination were performed prior to surgery for pre-operative surgical planning. All patients with primary diffuse (bilateral) CRS met consensus guidelines and were either classified as CRSwNP/eCRS (eosinophilic CRS) or non-eCRS by means of medical history, pre/intraoperative findings (i.e., surgeon reports polyps), and histological findings (i.e., polyp tissue ± eosinophilia) [6]. The study protocol was approved by the local ethics committee [(EK: 1760/2018)] and followed the Declaration of Helsinki on biomedical research involving human subjects. All patients provided written inform consent prior to participation.

### 2.2. Psychophysical Olfactory Testing

Orthonasal olfactory function was assessed in a well-ventilated room using the Sniffin’ Sticks test (Burghart Medical Technology, Wedel, Germany) [31,32]. The Sniffin’ Sticks test includes three subtests measuring odor threshold (n-butanol), discrimination, and identification. Odor threshold and discrimination performance were tested for each nostril separately, while identification was assessed birhinally. Unilateral orthonasal olfactory performance was calculated for each nostril separately by adding unilateral threshold and discrimination test scores with birhinal identification results (TDI left and TDI right). Clinically relevant side differences in orthonasal olfactory function were defined as TDI difference ≥ 6 points [12,33]. Birhinal orthonasal olfactory performance was calculated using the best-performing nostril method (TDI best) [12,13,15]. Based on the TDI best score, orthonasal olfactory function was classified as normal (TDI ≥ 30.75, normosmia), reduced (TDI < 30.75 and >16, hyposmia), or severely impaired (TDI ≤ 16, anosmia) compared to normative datasets [34].

Retronasal olfactory function was assessed using the Candy Smell Test (CST), which is validated in a 23-item version [35,36]. This study utilized the 27-item version [21], consisting of 27 sorbitol candies, each containing one individual aroma. Patients were instructed to suck or chew the candies and to identify the targeted aroma based on a four alternative forced choice procedure. Each correct answer yielded one point, resulting in 27 points to be achieved.

### 2.3. Radiological Evaluation

#### 2.3.1. Two-Dimensional Cross-Sectional Analysis

Uni- and bilateral OC-opacification was assessed based on an anterior, middle, and posterior two-dimensional coronal plane [26]: (i) the anterior section was specified at the anterior tip of the olfactory cleft, (ii) the middle section was defined at the posterior end of the ocular globe, and (iii) the posterior section was specified at the face of the sphenoid. Additionally, the medial boundary was defined at the superior attachment of the nasal septum for unilateral OC-opacification analysis. Unilateral and bilateral OC-opacification were then graded based on a Likert scale ranging from 0 to 3 [26]: 0 = 0 to 24%, 1 = 25 to 49%, 2 = 50 to 74%, and 3 = 75% to 100% opacification.

#### 2.3.2. Three-Dimensional Volumetric Analysis

**Figure 1:**
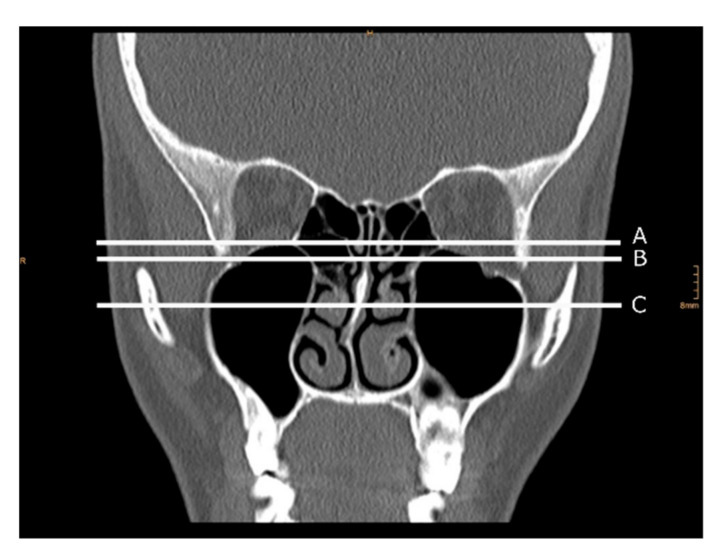
Caudal boundaries of the olfactory cleft (OC) used for OC-air volume analysis. The left side of the picture is the right side of the patient. (A) Horizontal line drawn 1 cm inferior to the cranial boundary. (B) Horizontal line drawn at the lower border of the superior turbinate; (C) horizontal line drawn at the head of the middle turbinate.

Olfactory cleft air volume analysis was performed using syngo.via (Siemens Healthcare Diagnostics, Erlangen, Germany). The range of −1024 to −200 Hounsfield units was defined as air volume. Air volume of the OC was assessed for each nostril separately. Overall OC-air volume was calculated as the sum of results from both nostrils. Boundaries of the OC were defined as previously reported [26]: (i) anterior: anterior plane of the middle meatus, (ii) lateral: attachment of the middle and/or superior turbinate, (iii) cranial: cribriform plate, (iv) caudal: horizontal line drawn 1 cm inferior to the cranial boundary (Air volume 1, Figure 1 (A)), and (v) medial: superior attachment of the nasal septum. Since olfactory receptor neurons can also be found in the area of the head of the middle turbinate [8], we defined two new caudal boundaries (in addition to the caudal boundary mentioned above): (a) horizontal line drawn at the lower border of the superior turbinate (Air volume 2, Figure 1 (B)) and (b) horizontal line drawn at the head of the middle turbinate (Air volume 3, Figure 1 (C)).

Additionally, CT scans were graded according to the Lund-Mackay Staging System and the depth of the olfactory fossa was classified according to the Keros classification [37,38]. An experienced radiologist (U.S.-N.), who was blinded to olfactory test results, performed all radiological analysis.

### 2.4. Statistical Analysis

Variables of interest were summarized as mean (standard deviation) or number (percentage). Assumption of normal distribution was assessed based on histograms. To compare olfactory test results and radiological findings of the OC between non-eCRS and CRSwNP, we used unpaired *t*-tests or the Mann–Whitney U test depending on the distribution. In a next step, we analyzed the association between bilateral OC-opacification (initially graded on a Likert-scale ranging from 0 to 3 = four groups) and birhinal orthonasal, and retronasal olfactory test results. To ensure adequate group sizes for this analysis (olfactory test results were stratified by Likert-scale OC-opacification groups), we merged olfactory test results from those with OC-opacification ranging from 0 to 24% (Likert-scale = 0) and 25 to 49% (Likert-scale = 1), and from those with OC-opacification ranging from 50 to 74% (Likert-scale = 2) and 75% to 100% (Likert-scale = 3) across all coronal planes to define two new groups: (i) partial OC-opacification (0% to 49%) and (ii) complete OC-opacification (50% to 100%). Likewise, we also merged unilateral orthonasal test results based on above-mentioned specifications to assess the relationship with ipsilateral OC-opacification. Olfactory results from both groups were then compared using unpaired *t*-tests. To determine whether OC-air volume was associated with olfactory test results, we performed bivariate correlation analysis between unilateral and bilateral OC-air volume with monorhinal and birhinal orthonasal, and retronasal test results. Correlation analysis were performed using Pearson’s correlation coefficient test. Results were considered significant where *p* < 0.05. GraphPad Prism 8.4.5 (GraphPad Software, Inc., La Jolla, CA, USA) was used for analysis and graphical visualization.

## 3. Results

### 3.1. Participants

**Figure 2:**
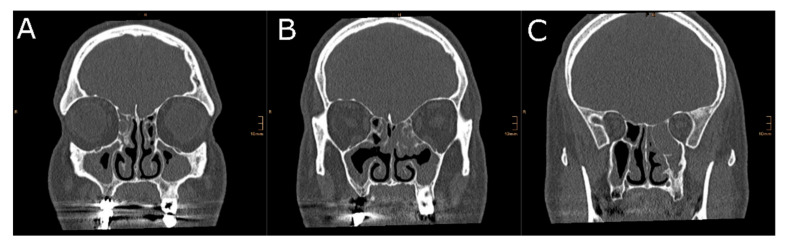
Two-dimensional cross-sectional analysis of the OC-cleft opacification in a patient with non-eosinophilic chronic rhinosinusitis. This patient had a TDI score of 29.5 at the right nostril, while olfactory testing of the left side revealed a TDI score of 22. The left side of the picture is the right side of the patient. (**A**) Anterior section, (**B**) middle section, and (**C**) posterior section.

Results from olfactory testing and radiological markers of the OC were analyzed in 28 patients (17 men, 11 women, mean ± SD age, 42.3 ± 14.3). Diagnosis included 13 CRSwNP, 12 non-eCRS, 1 septal deviation, and 2 asymmetric crooked noses (undergoing functional septorhinoplasty). We first divided our cohort into three groups: (i) 12 non-eCRS, (ii) 13 CRSwNP (iii), and 3 control, including our one patient with septal deviation and 2 with crooked noses. Olfactory test results revealed that 8 (28.6%) patients were normosmic, 15 (53.6%) hyposmic, and 5 (17.8%) functional anosmic. Clinically relevant side differences in orthonasal olfactory function were found in 3 (25%) of all non-eCRS and 3 (23.1%) of all CRSwNP cases (Figure 2). Orthonasal olfactory function (TDI best) was higher in non-eCRS compared to CRSwNP (29.4 vs. 21.7, *p* = 0.009). Similarly, OC-air volume based on all three caudal boundaries were greater in non-eCRS compared to CRSwNP (all *p* < 0.05). Likewise, group comparison of overall cross-sectional OC opacification across all three coronal planes revealed that the OC were more opacified in CRSwNP compared to non-eCRS (all *p* < 0.05) (Table 1).

### 3.2. OC-Opacification and Olfactory Test Results

**Figure 3:**
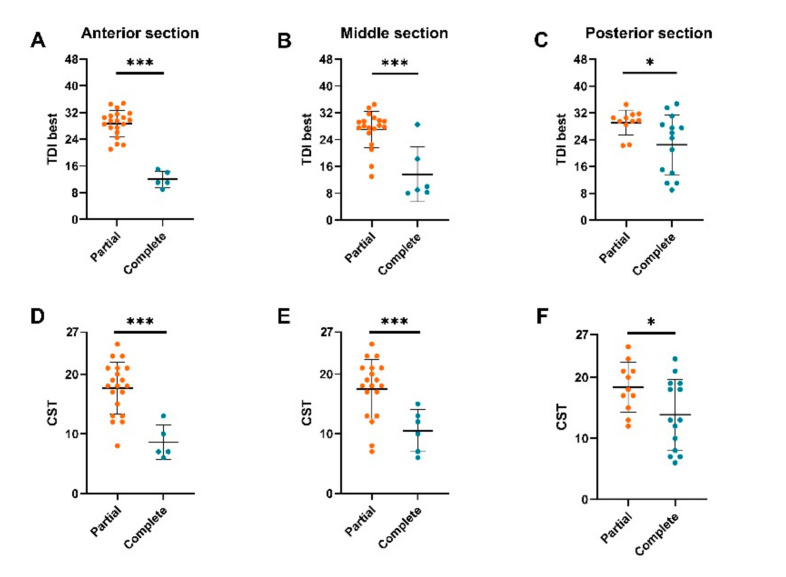
Birhinal orthonasal and retronasal olfactory function in chronic rhinosinusitis (CRS) patients with partial or complete obstruction of the olfactory cleft (OC). OC-opacification were assessed based on an anterior, middle, and posterior two-dimensional coronal plane. TDI best = birhinal orthonasal olfactory function, CST = retronasal olfactory function. OC-opacification assessed in the anterior section (**A**,**D**), middle section (**B**,**E**), and posterior section (**C**,**F**). *p < 0.05, ***p < 0.01.

**Figure 4:**
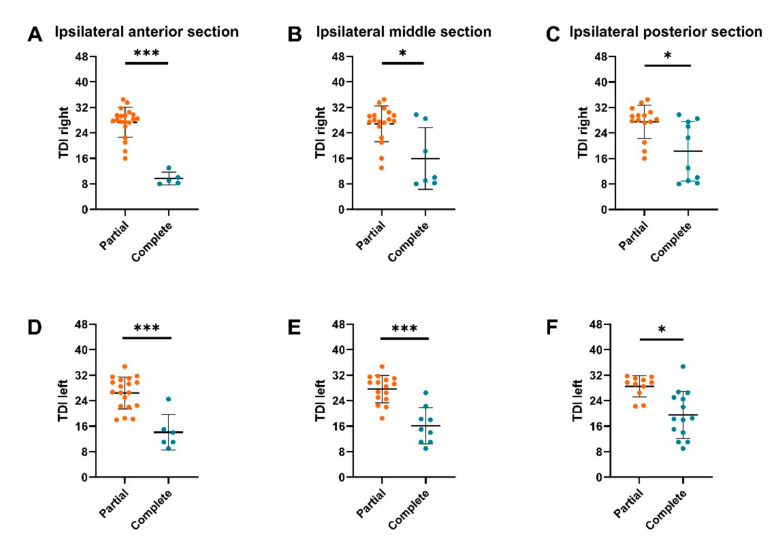
Monorhinal orthonasal olfactory function in CRS patients with partial or complete obstruction of the ipsilateral olfactory cleft (OC). Unilateral OC-opacification were assessed based on an anterior, middle, and posterior two-dimensional coronal plane. TDI left = orthonasal olfactory function of the left nostril, TDI right = orthonasal olfactory function of the right nostril. OC-opacification assessed in the ipsilateral anterior section (**A**,**D**), ipsilateral middle section (**B**,**E**), and ipsilateral posterior section (**C**,**F**). *p < 0.05, ***p < 0.01.

Analysis revealed significantly higher orthonasal and retronasal test results in CRS patients (*n* = 25) with partial OC-opacification compared to those with complete OC-opacification across all coronal planes (Figure 3). For the anterior coronal plane: total vs. partial (TDI best: 12.0 vs. 28.8, *p* < 0.001; CST: 8.6 vs. 17.7, *p* < 0.001), middle plane: total vs. partial (TDI best: 13.7 vs. 27.0, *p* = 0.001; CST: 10.5 vs. 17.5, *p* = 0.004), and posterior plane: total vs. partial (TDI best: 22.5 vs. 29.2, *p* = 0.030; CST: 13.9 vs. 18.4, *p* = 0.040). Similarly, unilateral orthonasal test results were also higher in those with ipsilateral partial OC-opacification compared to those with ipsilateral complete OC-opacification across all coronal planes of both nostrils (Figure 4). For the anterior coronal plane: right total vs. right partial (TDI right: 9.7 vs. 27.3, *p* <0.001), left total vs. left partial (TDI left: 14.1 vs. 26.5, *p* <0.001), for the middle coronal plane: right total vs. right partial (TDI right: 16.0 vs. 26.9, *p* = 0.002), left total vs. left partial (TDI left: 16.1 vs. 7.7, *p* < 0.001), for the posterior plane: right total vs. right partial (TDI right: 18.3 vs. 27.5, *p* = 0.004), left total vs. left partial (TDI left: 19.6 vs. 28.5, *p* = 0.001).

### 3.3. Associations with OC-Air Volume

**Figure 5:**
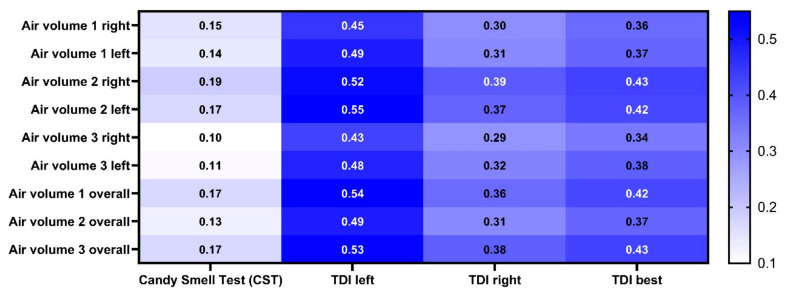
Correlation matrix between olfactory test results and olfactory cleft (OC)-air volume. The number represents Pearson’s r. Air volume 1 = the caudal boundary of the OC was defined at 1 cm inferior to the cranial boundary. Air volume 2 = the caudal boundary of the OC was defined at the lower border of the superior turbinate. Air volume 3 = the caudal boundary of the OC was defined at the head of the middle turbinate. TDI left = orthonasal olfactory function of the left nostril. TDI right = orthonasal olfactory function of the right nostril. TDI best = birhinal orthonasal olfactory function.

Correlation analysis revealed moderate positive correlations between birhinal orthonasal (TDI best) and overall air volume of the OC (r between 0.42 and 0.43, all *p* < 0.05). Significant positive correlations were also found between left sided TDI and ipsilateral air volume (r between 0.49 and 0.55, all *p* < 0.05). To the contrary, no relevant correlation was found between retronasal olfactory test results (CST) and overall OC-air volume or between right-sided TDI and ipsilateral OC-air volume (Figure 5).

## 4. Discussion

Previous studies provided first evidence for associations between overall OC-opacification and birhinal orthonasal olfactory function [23,24,25,26,27,28,29]. Nevertheless, there is a gap of knowledge with respect to the associations between unilateral radiological OC-markers with monorhinal orthonasal or retronasal olfactory function. In this study, we showed that higher OC-opacification was associated with lower orthonasal and retronasal olfactory function. We also showed that unilateral OC-opacification was associated with lower ipsilateral orthonasal olfactory function. Additionally, we found that monorhinal and birhinal orthonasal olfactory function correlated with ipsilateral and overall OC-air volume.

Together with previous works on the relationship between OC-opacification based on coronal plane analysis and orthonasal olfactory function, our results confirm that higher OC-opacification can be associated with lower orthonasal olfactory function [23,24,25,26,28]. Furthermore, we showed that higher unilateral OC-opacification was associated with lower ipsilateral orthonasal olfactory function. More importantly, we hypothesized that higher OC-opacification might also be associated with lower retronasal olfactory function (as measured using the CST), which we found to be the case—regardless of odorants coming from the back route. Furthermore, differences in orthonasal and retronasal olfactory function were most pronounced in patients that were stratified based on OC-opacification analysis using the anterior-most coronal plane.

In particular, the latter finding may be of high clinical relevance as it can be implemented effortless into clinical routine: simply assessing the opacification of the OC in one section based on two-dimensional coronal planes (of the most anterior section) may already provide valuable insight into unilateral and bilateral orthonasal, and retronasal olfaction. Ortho- and retronasal olfactory function can be impaired in OD patients with opacified OC. This has to be taken into account due to the significant impact of smell loss—and especially loss of flavor perception—on QOL of affected individuals [39,40].

Prior studies on radiological OC-markers in patients with CRS revealed that birhinal orthonasal olfactory function correlated negatively with OC-opacification based on air volume analysis [25,26]. Since volatile odor molecules approach the olfactory receptors through the air, one might reasonably suggest that overall air volume of the OC also correlates positively with olfactory function, independent from the route of presentation (i.e., ortho- or retronasally). In our study, we found that OC-air volume was correlated positively with overall orthonasal olfactory function. More importantly, lateralized orthonasal olfactory function also showed positive correlations with ipsilateral OC-air volume. To the contrary, no relevant correlation was found between retronasal olfactory function and OC-air volume. One explanation for this finding might relate to the differences in airflow rates between orthonasal (during inspiration/sniffing) and retronasal olfaction (during expiration, mastication, and deglutition) [41]. Fluid dynamic models of retronasal odor transportation provided evidence that odor volatiles are most effectively transported into the nasal cavity during quiet breathing and therefore might be less dependent on the actual air volume of the OC.

The current study utilizes results from comprehensive lateralized orthonasal and retronasal smell tests to investigate the effect of radiological OC-markers on olfactory function. Nonetheless, some limitations of this study merit consideration. First, we included a relatively small sample of patients and controls and therefore our results come with the associated limitations. Future studies will need to include larger sample sizes of patients and control subjects to verify current findings. Additionally, this was a cross-sectional study while a longitudinal study would more directly capture the shift in olfactory function over time in CRS patients. A longitudinal study design might also allow to depict a possible causal relationship between the OC-opacification and findings from olfactory testing, More specifically, considering the (chronic) inflammation of the olfactory epithelium in patients with CRS [42], it might be hypothesized that some patients will not regain olfactory function after endoscopic sinus surgery, as the reason for their smell loss is not “conductive” (in cases of edema or nasal polyps), but rather a damage to the “sensorineural” pathway within the olfactory epithelium. Last, although we found radiological findings to be of predictive value for olfactory function in this cohort, semi-objective olfactory testing by means of validated smell tests remains mandatory. Especially in cases of litigation, or whenever the exact extent of chemosensory function is clinically demanded for reasons of counseling, comprehensive olfactory testing should be pursued.

## 5. Conclusions

Analyzing existing and novel radiological markers and comprehensive olfactory test scores, we found higher OC-opacification to be associated with lower lateralized and overall orthonasal, and retronasal olfactory function. Moreover, lateralized and overall orthonasal olfactory function correlated with OC-air volume, in contrast to retronasal odor identification. These findings add to the current literature with a strong potential for clinical implementation.

## Figures and Tables

**Table 1:** Demographic, olfactory, and radiologic characteristics. Data are presented as mean (standard deviation) or number (%). TDI = Sniffin’ Sticks TDI test, CST = Candy Smell Test.

Demographics					
	CRS	Non-eCRS	CRSwNP	*p* Value	Control
Age in years	42.1 (14.3)	40.3 (14.3)	43.5 (14.1)	0.77	38.9 (14.0)
Gender	9F, 16 M	5F, 7M	4F, 9M	-	2F, 1M
**Olfactory characteristics**					
TDI best	25.4 (7.6)	29.4 (3.7)	21.7 (8.3)	0.01	30.3 (4.1)
Left	23.5 (7.2)	27.7 (4.6)	19.6 (7.0)	<0.01	29.8 (5.0)
Right	23.8 (8.2)	27.5 (4.9)	20.4 (9.1)	0.03	28.5 (3.5)
Threshold					
Left	3.9 (2.6)	4.9 (2.4)	3.0 (2.4)	0.07	6.8 (1.4)
Right	4.1 (2.5)	4.6 (2.1)	3.7 (2.7)	0.39	4.8 (1.0)
**Discrimination**					
Left	8.9 (2.7)	10.3 (2.7)	7.6 (2.1)	0.02	9.7 (2.1)
Right	9.0 (2.9)	10.3 (1.8)	7.7 (3.2)	0.03	10.3 (2.1)
Identification	10.7 (4.1)	12.6 (2.0)	9.0 (4.7)	0.03	13.3 (1.7)
CST	16.0 (5.4)	17.9 (3.2)	14.1 (6.4)	0.09	18.0 (5.1)
Normosmics	6 (24%)	5 (41.7%)	1 (7.7%)	-	2 (66.7%)
Hyposmics	14 (56%)	7 (58.3%)	7 (53.8%)	-	1 (33.3%)
Anosmics	5 (20%)	0	5 (38.5%)	-	-
**Olfactory Cleft Opacification**					
Opacification					
Left side					
Anterior section	0.7 (1.2)	0.0 (0.0)	1.3 (1.4)	0.02	0.0 (0.0)
Middle section	1.2 (1.2)	0.6 (1.0)	1.7 (1.1)	0.01	0.7 (0.9)
Posterior section	1.6 (1.4)	0.8 (1.3)	2.5 (0.9)	0.01	0.0 (0.0)
Right side					
Anterior section	0.6 (1.2)	0.0 (0.0)	1.2 (1.4)	0.02	0.0 (0.0)
Middle section	0.9 (1.2)	0.5 (1.0)	1.2 (1.3)	0.15	0.7 (0.9)
Posterior section	1.3 (1.4)	0.4 (0.9)	2.1 (1.3)	<0.01	0.0 (0.0)
Overall					
Anterior section	0.6 (1.2)	0.0 (0.0)	1.2 (1.4)	0.02	0.0 (0.0)
Middle section	1.1 (1.1)	0.6 (0.9)	1.5 (1.1)	0.02	0.7 (0.9)
Posterior section	1.5 (1.3)	0.7 (0.9)	2.3 (1.0)	<0.01	0.0 (0.0)
**Olfactory Cleft Air Volume (cm^3^)**					
Air Volume 1					
Left	0.5 (0.4)	0.7 (0.4)	0.4 (0.3)	0.05	0.8 (0.1)
Right	0.5 (0.3)	0.6 (0.3)	0.4 (0.3)	0.08	0.8 (0.2)
Overall	1.2 (0.8)	1.5 (0.7)	0.9 (0.6)	0.03	1.9 (0.2)
Air volume 2					
Left	0.7 (0.5)	0.9 (0.5)	0.5 (0.4)	0.03	1.1 (0.0)
Right	0.7 (0.5)	0.9 (0.4)	0.5 (0.4)	0.04	1.1 (0.1)
Overall	0.9 (0.6)	1.2 (0.6)	0.7 (0.5)	0.05	1.5 (0.3)
Air volume 3					
Left	0.4 (0.3)	0.6 (0.3)	0.3 (0.2)	0.03	0.7 (0.2)
Right	0.4 (0.3)	0.5 (0.3)	0.3 (0.3)	0.07	0.7 (0.2)
Overall	1.1 (0.7)	1.4 (0.7)	0.8 (0.6)	0.03	1.8 (0.2)
